# Implications of gendered behaviour and contexts for social mobility in the USA: a nationally representative observational study

**DOI:** 10.1016/S2542-5196(19)30191-3

**Published:** 2019-10-01

**Authors:** Benjamin W Domingue, Beniamino Cislaghi, Jason M Nagata, Holly B Shakya, Ann M Weber, Jason D Boardman, Gary L Darmstadt, Kathleen Mullan Harris

**Affiliations:** **Graduate School of Education and Population Health Sciences, Stanford University, Stanford, CA, USA** (B W Domingue PhD)**; Department of Global Health and Development, London School of Hygiene & Tropical Medicine, London, UK** (B Cislaghi PhD)**; Department of Pediatrics, University of California, San Francisco, CA, USA** (J M Nagata MD)**; Center on Gender Equity and Health, Division of Infectious Disease and Global Public Health, University of California, San Diego, CA, USA** (H B Shakya PhD)**; School of Community Health Sciences, University of Nevada, Reno, NV, USA** (A M Weber)**; Institute of Behavioral Science and Department of Sociology, University of Colorado, Boulder, CO, USA** (Prof J D Boardman)**; Department of Pediatrics, Stanford University School of Medicine, Stanford, CA, USA** (Prof G L Darmstadt)**; and Carolina Population Center and Department of Sociology, University of North Carolina, Chapel Hill, NC, USA** (Prof K M Harris PhD)

## Abstract

**Background:**

We constructed measures of an individual’s gendered behaviour and their gendered environment to investigate the salience of gender norms during adolescence for social mobility during the next decade of life.

**Methods:**

In this nationally representative observational study, we collected individual-level data from the National Longitudinal Study of Adolescent to Adult Health (Add Health), which enrolled a cohort of nationally representative school students aged 11–19 years from across the USA and followed them up for 14 years (ie, to age 25–33 years). We characterised gendered behaviour for adolescents in a performative sense via self-reports of behaviours and beliefs. We aggregated this individual-level measure to create a proxy measure of an individual’s social context by taking averages for an individual’s peers of the same sex and school year.

**Findings:**

Between Jan 5, 1994, and Dec 26, 1995, Add Health collected data on a cohort of 20 745 students. 14 540 respondents were followed-up 14 years later between April 3, 2007, and Feb 1, 2009, of whom 7722 (53·1%) were female. More masculine male respondents were downwardly mobile; they were enrolled in school for fewer years and were more likely to have lower status jobs than their less masculine same-sex school peers. More masculine male respondents were also more likely to have jobs in occupational categories with larger proportions of males than their same-sex school peers. Gendered behaviour was not predictive of future educational and occupational attainment for female respondents. Male adolescents in school years with more masculine same-sex peers than male adolescents in other school years also tended to have lower educational and occupational attainment than their male peers. Educational and occupational attainment in early midlife for female respondents was not affected by their gendered environment.

**Interpretation:**

Gender, when measured as a set of gender-distinct behaviours in adolescence, was associated with differential patterns of social mobility from adolescence to young adulthood. Moreover, variation in an individual’s local gender norms has implications for subsequent socioeconomic attainment, especially for male adolescents. These findings have potential implications for observed health disparities.

**Funding:**

Bill & Melinda Gates Foundation.

## Introduction

Gender is a core feature of the human experience, combining both psychological and social components. Gender identity—including performative aspects^1^—has psychological implications for the ways in which an individual interacts with the world. Beyond identity, gender constitutes a system of norms that shape acceptable actions for people who are perceived as being of a given gender (eg, norms regarding so-called appropriate clothing for men and women). Gender norms are a pivotal component of an individual’s social environment, especially during crucial developmental periods—such as adolescence^2,3^—these norms might have far-reaching effects.^4,5^ Some effects will manifest in contemporaneous behaviour while others might manifest later in life. Evidence suggests that social environments, especially ecological measures of socioeconomic status,^6^ have such effects during this period. Beyond socioeconomic status, measures of local norms and an individual’s concordance with those norms have implications for their subsequent development and wellbeing.^5^ These norms can potentially be measured through longitudinal survey data.^7–9^ Little is known about the implications of differential exposures to gender norms for an individual’s long-term outcomes. Given global commitments to advancing gender equality—eg, as captured in the UN Sustainable Development Goal (SDG) 5^10^—such research is crucial.

Here, we build on previous efforts to construct measures of an individual’s gendered behaviour^11^ and a broader measure of an individual’s gendered environment^12^ to investigate the salience of gender norms during adolescence. Specifically, we investigated the implications for socioeconomic attainments a decade later in life. Such an understanding is important because differences in gender-related norms between places (eg, geographical locations)^13,14^ could have implications for individuals’ subsequent patterns of socioeconomic attainment—a key social determinant of health.^15^ Findings from a study that used data from the US National Longitudinal Study of Adolescent to Adult Health (Add Health) suggest that those who have discrepancies between their gender-discriminating behaviours and beliefs are at higher risk of a variety of health-related effects than their peers of the same sex in the same school grade (hereafter referred to as school year for international generalisability).^5,7^ These findings, along with other evidence,^16^ suggest that the gendered environment might have public health implications; however, implications for broader patterns of life course attainments are not known. Although gender socialisation might occur at many different levels,^17^ here we focus on schools. Adolescence is a unique developmental period in many respects^18^ and, during this period, schools are a crucial social environment.^19^

**Research in context**

**Evidence before this study**

Recent evidence suggests that gendered behaviour and environments can affect an individual’s health. However, little is known about the implications of gendered behaviour and environment on an individual’s socioeconomic attainments. Given strong observed health disparities as a function of socioeconomic attainments, gender could have long-term implications for health.

**Added value of this study**

Our analyses suggest that gendered behaviour is associated with downward social mobility relative to an individual’s schoolmates for male adolescents—ie, more masculine male adolescents tend to be in school for fewer years and have less prestigious occupations. We observed no such trends for female adolescents. We also observe associations between gendered environments and subsequent attainments for male adolescents. Those with more masculine same-sex school-year peers tend to have lower socioeconomic attainments compared with their school peers in different school years. Female adolescents are relatively resilient, with their attainments in later life being independent of the gendered behaviour of their same-sex school-year peers.

**Implications of all the available evidence**

Our findings suggest that the gender norms experienced by an adolescent could have long-term implications on their socioeconomic position, especially for male adolescents. Efforts should be made to refine our understanding about the specific features of gendered environments that might hinder individual wellbeing.

In this study, we aimed to understand the nature of gendered social contexts for US adolescents and their consequences on socioeconomic status more than 10 years later. Given previous findings related to health, we anticipate that gendered behaviour and context in adolescence might have implications for subsequent socioeconomic attainments. We first examined the broader features of individuals and environments that covary with our measures of gendered behaviour. Specifically, we looked at associations between an individual’s behaviour and background and their gendered behaviour, and associations between environmental characteristics and the gendered context of an individual’s social environment (eg, their school). Next, we investigated the downstream correlates of an individual’s gendered behaviour specifically examining the types of socioeconomic attainments in early midlife (ie, age 25–33 years) predicted by gendered behaviour in adolescence. Finally, we looked at the potential implications of gender norms among a relatively exogenous group of social peers—an individual’s (same-sex) school-year peers. In particular, we assessed the implications of exposure to relatively masculine or feminine same-sex school-year peers on later socioeconomic attainments compared with what would be expected for an individual within a given school.

## Methods

### Study population

In this nationally representative observational study, we collected individual-level data from Add Health,^20^ a nationally representative cohort drawn from a probability sample of US schools in roughly 80 US communities, representative of schools in the USA for the period 1994–95 with respect to region, urban setting, school size, school type, and race or ethnic background. Add Health collected data on adolescents aged 11–19 years (wave 1) and followed them up 14 years later (wave 4) when they were aged 25–33 years. Data were collected via interview and the interveiwer recorded the sex of each respondent. We focused on measures of gender normative behaviour and context from wave 1 and outcomes from wave 4.

### Measures

The measures we used in this study are gendered behaviour and gendered context. More detail on these measures is in the appendix (pp 1–2), but we will describe them here briefly. We measured an individual’s gendered behaviour on the basis of self-reports regarding behaviours (eg, wearing a seatbelt) and beliefs (eg, feels that friends care about them). To measure an individual’s gendered context, we used the aggregate gendered performances of an individual’s same-sex school-year peers.

**Figure 1:**
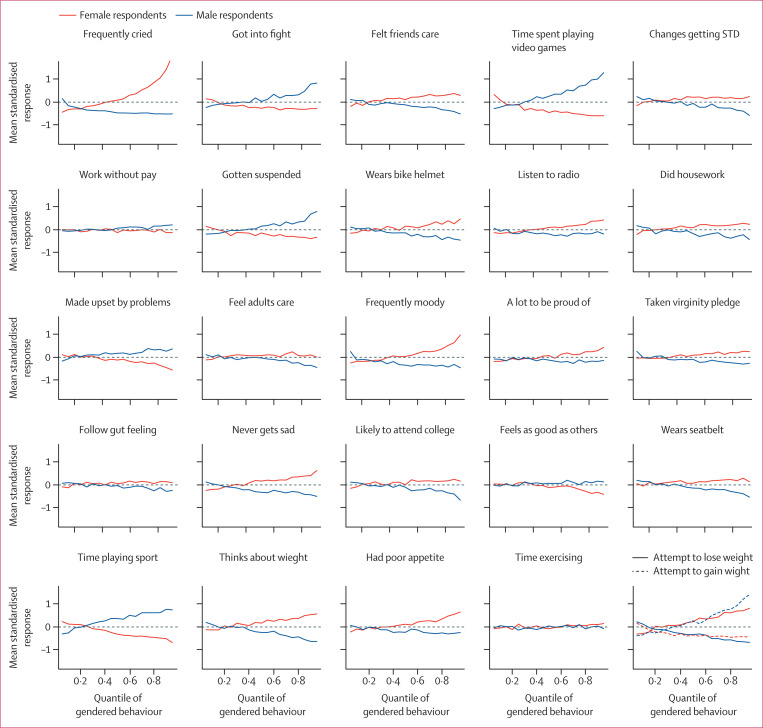
Mean for each standardised variable as a function of the constructed gendered behaviour variable, by sex For each quantile from 0·05 to 0·95 (in increments of 0·05), all respondents of the appropriate sex whose gendered behaviour value is within 0·025 of the specified quantile are combined; the variable mean is then taken over this combined group. STD=sexually transmitted disease.

We constructed our key measure of gendered behaviour using a set of variables identified in previous work.^11^ For a given school, we first fit a logistic regression model based on 26 variables (detailed in the appendix [pp 6–7]) in which the outcome is sex at wave 1 using only data from schools other than the school of interest. We then used these estimated coefficients to predict the probability of sex for each respondent in the focal school. Using these predicted probabilities, we focused on the probability of a respondent being their sex as identified by Add Health interviewers (ie, lower values can be interpreted as higher levels of gender-atypical behaviour; higher values as more gender-typical behaviour). Distributions for this variable look similar for male and female respondents (appendix p 15). Generally, gender-atypical adolescents (more masculine female adolescents and more feminine male adolescents) behave similarly irrespective of sex; differences between sexes typically emerge at larger quantiles in the distribution (figure 1). For instance, time spent playing video games, gender-atypical adolescents (quantiles <0·4) of both sexes play video games at a similar frequency. However, for gender-typical adolescents (quantiles >0·6), male adolescents spend far more time playing video games than do female adolescents.

For the measure of gendered context, for each respondent we calculated means of the gendered behaviour of the same-sex school peers of the individual of interest in the same school year. Generally, we found a negative correlation between the school-level and school-year-level aggregates of gendered behaviour across genders (appendix p 15). This finding suggests that, within a social environment, behaviour tends to be more masculine or more feminine across sexes (rather than more sex-concordant). Within a school, we tend to observe all respondents being either more masculine or more feminine rather than observing male respondents being more masculine and female respondents being more feminine. We also note substantial within-school heterogeneity in the school-year-level measures of gendered behaviour (appendix p 16). We used this variation as a point of leverage. Along nearly any dimension, differences between schools are far from random (eg, schools are in different sociopolitical climates in different states). However, in a given school, differences between school years are potentially driven by exogenous differences and show a reduced influence of structural factors.

### Analysis of wave 1 and wave 4 data

Of the data collected in wave 1 of Add Health, we considered a variety of individual-level measures to characterise correlates of adolescent gendered behaviour, including the following: verbal ability via a modified version of the Peabody vocabulary test,^21^ high-school grade-point average (GPA; collected from transcripts upon completion of school),^22^ household socioeconomic status,^23^ and a cumulative contextual measure of neighbourhood disadvantage generated via a link between student home address and US census data.^24^ Given the potential for associations between an individual’s religious beliefs and their beliefs about gender roles,^25^ we also used an indicator of the textual inerrancy of the core texts of their religious faith. We also considered aggregates of these measures and additional measures defined solely at the school level (eg, characteristics of the distribution of parental education within a school). Particularly, we considered two measures derived from data on maternal education from the entire set of students at each school observed during wave 1: the proportion of mothers who graduated high school and inequality in the distribution of education among these mothers, as measured using the Gini coefficient.^26^

Of the data in wave 4, we focused on a variety of socioeconomic attainments measured when respondents were aged 25–33 years. We used a measure of educational attainment (number of years of schooling) and a measure of occupational status^23^ based on the typical income and education level of members of that occupational group. On the basis of the respondent’s job code, we linked the job categories of Add Health respondents to federal data to determine the share of women in the same occupational category (more details are in the appendix [p 2]). We also focused on a measure of neighbourhood disadvantage based on where the respondent resided in wave 4.^24^ Finally, we compared those respondents who were not in education, employment, or training with those who were undertaking one of these activities.^27^ Additional details on all measures are in the appendix (pp 1–2).

### Statistical analysis

To better understand the nature of our gendered behaviour variable, we first examined correlations between gendered behaviour (at the individual level and school level) and a variety of individual-level and school-level variables (eg, socioeconomic status and GPA). We then examined associations between gendered behaviour and socioeconomic outcomes measured 14 years later. To test for monotonicity, we did a locally weighted smoothing (LOESS) analysis wherein socioeconomic outcomes (residualised for birth year and school fixed effects) were examined as a function of gendered behaviour. Given that we generally observe monotonic trends, we then undertook linear regression analyses.

Our main analyses were based on standardised association between outcomes at wave 4 and gendered behaviour at wave 1. For a given outcome (*y*; standardised in the full sample before analyses) and focal variable (*x*; gendered behaviour or context), we used a model with school as a fixed effect of the form *y_ij_* = *b*_0_ + *b*_1_*x_ij_* + controls + μ*_j_* + e*_ij_*, in which *i* indexes individuals clustered in a school, *j, b*_0_ and *b*_1_ are regression coefficients, e is the regression error term, and μ is the school-level fixed effect. We focused on results based on school fixed-effect analysis since it allows for interrogation of the associations between behaviour and outcomes compared with an individual’s schoolmates (ie, across years). We did our analyses separately by sex and they are weighted to make the wave 4 sample comparable with the wave 1 sample;^28^ SEs are clustered at the school level. For analyses of an individual’s gendered behaviour, we included controls for an individual’s birth year, race and ethnicity, and household socioeconomic status. Our primary interest is on the association between *y* and *x* as measured by the estimate *b*_1_ (ie, the standardised association). We also examined associations between gendered behaviour and sexual behaviour (appendix pp 2–3).

We examined associations with gendered context using a similar analytical approach. To test for monotonicity, we did a LOESS analysis. We then did linear regression analyses using a variant of the above equation. In particular, for analyses of gendered context, we also included the individual’s gendered behaviour as a control. We also analysed associations between an individual’s wave 4 outcomes and the gendered context defined by their opposite-sex school-year peers.

In a secondary analysis, we considered a small set of variables related to key behaviours with implications for the labour market and feelings of social status (appendix p 2).

We undertook several ancillary analyses. We did analyses to investigate heterogeneity as a function of racial and ethnic group membership. We tested for non-linearities in associations between gendered behaviour and occupational and educational attainment. We investigated the effect of measurement error in our measure of gendered behaviour on our findings. We also did an analysis on a subsample that did not contain single-sex schools (or schools with substantial imbalance in the distribution of male and female students).

### Role of the funding source

The funder had no role in study design, data collection, data analysis, data interpretation, or writing of the report. The corresponding author had full access to all the data in the study and final responsibility for the decision to submit for publication.

## Results

In the Add Health cohort,^20^ data were collected between Jan 5, 1994, and Dec 26, 1995 (wave 1), for 20 745 students aged 11–19 years, of whom 14 540 (70·1%) were female. This cohort was followed up 14 years later, between April 3, 2007, and Feb 1, 2009 (wave 4), and data were collected for 14 540 respondents aged 25–33 years, of whom 7722 (53·1%) were female. Based on self-reports, 56% of respondents were white, 21% were black, 6% were Asian, and 15% were Hispanic.

An individual’s gendered behaviour is weakly associated (|r| <0·15; table 1) with their verbal ability and school performance (as measured via high-school GPA). In both male and female respondents, more masculine behaviour is associated with lower household socioeconomic status and higher neighbourhood disadvantage. This patterning of gendered behaviour across an individual’s social origin is a clear source of confounding in analyses of socioeconomic attainments in later waves of data collection, and an issue we attempted to address through inclusion of socioeconomic attainment as a control and a focus on within-school comparisons (ie, school fixed-effect analysis). Gendered behaviour is not associated with an individual’s belief in the inerrancy of their core religious texts. We also found sexual behaviour to be strongly patterned as a function of gendered behaviour (appendix p 18).

**Table 1:** Individual-level and school-level correlations between wave 1 gendered behaviour and individual-level correlates and the broader gendered environment and ecological correlates

	Female respondents		Male respondents	
**Individual-level correlations**				
Peabody vocabulary test	0·07 (0·05 to 0·09)		–0·05 (–0·07 to –0·02)	
Overall GPA	0·09 (0·06 to 0·11)		–0·14 (–0·17 to –0·12)	
Mathematical GPA	0·08 (0·06 to 0·11)		–0·14 (–0·17 to –0·11)	
Household socioeconomic status	0·07 (0·05 to 0·10)		–0·08 (–0·11 to –0·06)	
Neighbourhood disadvantage	–0·10 (–0·12 to –0·07)		0·08 (0·06 to 0·10)	
Inerrant religious text	0·00 (–0·02 to 0·03)		–0·01 (–0·03 to 0·01)	
**School-level variables**				
Peabody vocabulary test	0·40 (0·25 to 0·53)		–0·24 (–0·39 to –0·07)	
Household socioeconomic status	0·25 (0·09 to 0·40)		–0·22 (–0·37 to –0·06)	
Neighbourhood disadvantage	–0·22 (–0·37 to –0·06)		0·23 (0·06 to 0·38)	
Inerrant religious text	–0·09 (–0·25 to 0·08)		–0·17 (–0·32 to –0·01)	
Proportion of mothers who graduated high	–0·06 (–0·23 to 0·12)		0·09 (–0·09 to 0·27)	
school				
Gini coefficient in years of mother’s education	0·13 (–0·05 to 0·30)		–0·24 (–0·40 to –0·06)	

Data are correlation coefficients and 95% CI in parentheses. Gender designations as made by Add Health at wave 1. GPA=high-school grade-point average

Aggregated to the school level, gendered context is strongly associated with a variety of ecological measures (table 1). Schools where male adolescents tend to have highly masculine behaviours have lower mean Peabody score and household socioeconomic status and higher levels of neighbourhood disadvantage—as would be expected given the individual-level correlations. Schools with female adolescents with highly feminine behaviours have higher Peabody scores and household socioeconomic status and lower levels of neighbourhood disadvantage. Schools with students that have relatively well educated mothers are likely to have female students who engage in gender-typical (feminine) behaviours but males who do not engage in gender-typical (masculine) behaviours.

**Figure 2:**
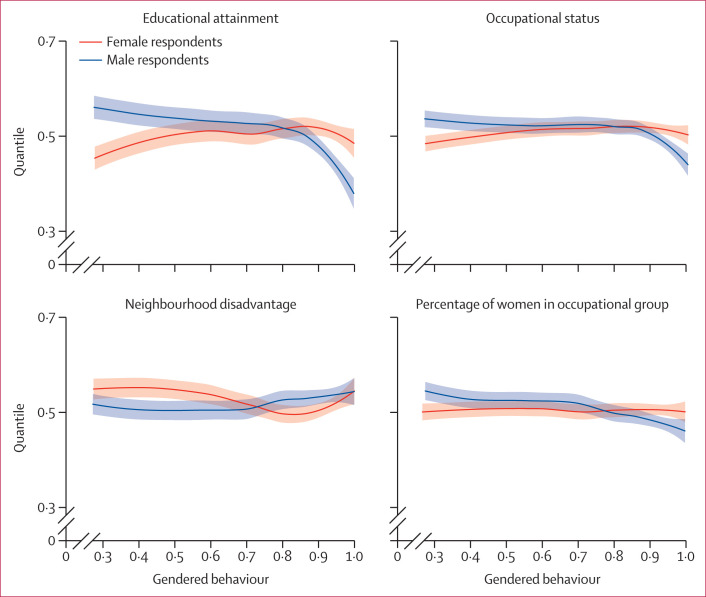
Associations between an individual’s gendered behaviour in wave 1 and their outcomes in wave 4, residualised for birth year and a school fixed effect The y-axes extend from the 0·3 quantile to 0·7 quantile of the residualised outcome distribution. The solid lines are the association and shaded areas are 95% CIs.

Gendered behaviour of male adolescents in wave 1 is strongly predictive of socioeconomic attainments at wave 4. In descriptive analyses, more masculine male adolescents tend to have relatively lower educational attainment then their less masculine male peers and more feminine female adolescents tend to have relatively higher education attainment than their less feminine female peers (figure 2). This trend is less pronounced for female respondents than for male respondents. We found a qualitatively similar pattern for occupational status but the effects are weaker than for educational attainment. More masculine men are more likely to have jobs with relatively few women than those who are less masculine, whereas gendered behaviour is not predictive of the sex composition of the occupational group for women. Gendered behaviour was largely unassociated with neighbourhood disadvantage.

In more formal tests (table 2), we looked at associations between gendered behaviour in wave 1 and outcomes in wave 4 residualised for birth year, race and ethnicity, household socioeconomic status, and a school fixed effect. More masculine behaving male adolescents tend to have fewer years of education (*b*_1_ = –0·280; p<0·0001) and lower occupational status (*b*_1_ = –0·178; p=0·0091) by early midlife than their same-sex schoolmates from adolescence. The lower occupational status of more masculine men than their same-sex schoolmate peers was largely mediated by educational attainment (appendix p 14). By contrast, gendered behaviour of women does not predict future educational or occupational attainment. Male adolescents who exhibited more gender-typical (masculine) behaviour in wave 1 were also likely to be employed in an occupation with a lower percentage of women (*b*_1_ = –0·209; p=0·0016; table 2). This association might act as a potential explanatory mechanism for the lower occupational status among more masculine men; among the male respondents, we found an association between occupational status and the proportion of women in that occupational group (*r* = 0·3; and among female respondents, *r* = –0·07).

**Table 2:** Standardised associations between individual gendered behaviour and gendered environment as identified by same-sex school-year peers for wave 1 and wave 4 outcomes

	**Individual gendered behaviour**		**Gendered behaviour of same-sex** **school-year peers**	
	Female respondents	Male respondents	Female respondents	Male respondents
**Educational attainment**				
n	6936	6212	6860	6097
*b*_1_	0·069 (–0·042 to 0·180)	–0·280 (–0·398 to –0·163)	0·517 (–0·002 to 1·036)	–1·036 (–1·553 to –0·519)
p value	0·23	<0·0001	0·05	<0·0001
**Occupational status**				
n	6791	6043	6721	5932
*b*_1_	0·032 (–0·103 to 0·168)	–0·178 (–0·312 to –0·044)	0·386 (–0·228 to 0·999)	–0·848 (–1·381 to –0·315)
p value	0·64	0·0091	0·22	0·0018
**Neighbourhood disadvantage**				
n	6876	6156	6800	6043
*b*_1_	0·033 (–0·074 to 0·139)	0·003 (–0·132 to 0·138)	–0·166 (–0·660 to 0·327)	0·233 (–0·389 to 0·855)
p value	0·55	0·97	0·51	0·46
**Not in education, employment, or training**				
n	6937	6213	6861	6098
*b*_1_	–0·013 (–0·154 to 0·129)	0·095 (–0·047 to 0·237)	–0·371 (–1·265 to 0·523)	0·413 (–0·188 to 1·013)
p value	0·86	0·19	0·42	0·18
**Percentage of women in occupational group**				
n	6435	5361	6370	5260
*b*_1_	–0·008 (–0·127 to 0·111)	–0·209 (–0·339 to –0·080)	–0·409 (–0·905 to 0·087)	–0·659 (–1·204 to –0·113)
p value	0·90	0·0016	0·11	0·018

Data are n, and standardised associations (*b*_1_) calculated using a school fixed-effect model with 95% CIs in parentheses and p values. For individual gendered behaviour, estimates are net of birth year, race and ethnicity, adolescent socioeconomic status, and a school fixed effect. For gendered environment, estimates are net of an individual’s gendered behaviour, birth year, race and ethnicity, adolescent socioeconomic status. and a school fixed effect. Analyses are based on individuals without missing data.

We explored heterogeneity in the findings as a function of race and ethnicity (appendix p 3) but note that interpretation is challenging given small sample sizes. Focusing on male respondents, findings pertaining to educational attainment are relatively stable across racial and ethnic groups (appendix p 19). Motivated by the associations we found between gendered behaviour in wave 1 and outcomes in wave 4 (figure 2), we also tested for non-linearities in the association between gendered behaviour and educational and occupational attainment. We found that these results are driven largely by the most masculine male respondents (appendix p 20). We found measurement error in our measure of gendered behaviour to have some small effect on the significance of results but key findings were robust (appendix p 12). Finally, when analysing schools with a relatively even gender split, three schools were removed from the analysis, and the results were similar to the main analysis results (appendix p 13).

We also investigated associations with additional wave 4 outcomes related to key behaviours with implications for the labour market and feelings of social status (appendix p 10). We first investigated outcomes with implications for the labour market—ie, military enlistment and involvement with the criminal justice system. More masculine male respondents are more likely to both enlist in the military and be involved in the criminal justice system than their same-sex school peers. We observed no such association for female respondents. We then investigated two measures of an individual’s perception of social status and integration that present an interesting set of caveats to the above findings. More masculine male respondents feel less isolated than their same-sex school peers and they also have no decrease in their perceived social standing relative to their peers when asked to place themselves on a respect ladder. By contrast, more feminine female respondents feel more isolated and less respected than their same-sex school peers.

**Figure 3:**
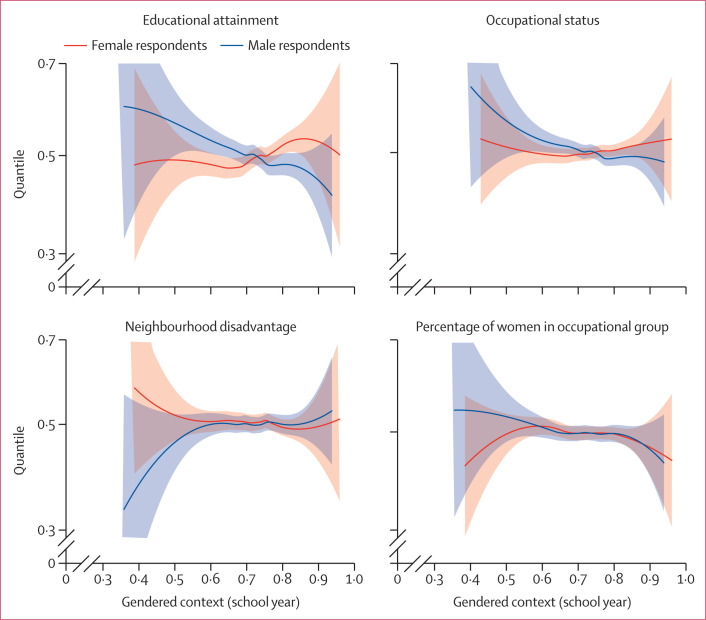
Associations between gendered context in wave 1 and outcomes in wave 4, residualised for an individual’s own gendered behaviour, birth year, and a school fixed effect Gendered context is identified via the behaviour of an individual’s same-sex school-year peers. The y-axes extend from the 0·3 quantile to 0·7 quantile of the residualised outcome distribution. The solid lines are the association and shaded areas are 95% CIs.

We also investigated associations between gendered context and social mobility. More masculine adolescent social environments (as proxied by the respondent’s same-sex school-year peers) also predict reduced socioeconomic attainments for male respondents in early midlife. Descriptive results (figure 3) are qualitatively similar to those in figure 2. Formal tests considered outcomes controlling for gender behaviour of school-year peers, own gendered behaviour, birth year, race and ethnicity, household socioeconomic status, and a school fixed effect (table 2). Compared with their same-sex schoolmates, male respondents whose school-year peers showed increasingly gendered behaviour tended to have fewer years of educational attainment (*b_1_*=–1·036; p<0·0001), and for those employed, lower occupational status (*b_1_* =–0·848; p=0·0018). More masculine school-year peers are also potentially predictive of jobs in occupational categories with fewer women (*b_1_*=–0·659; p=0·018); whereas, the gender composition of occupational categories for female respondents is not affected by the gendered behaviour of their same-sex school-year peers. Gendered context is not associated with individuals not being in education, employment, or training for either sex.

We also considered the association between wave 4 outcomes and the gendered context of an individual’s opposite-sex school-year peers. Generally, associations were weak (appendix p 11), suggesting that specifically the behaviour of an individual’s male classmates potentially leads to affective and behavioural changes associated with differences in long-term occupational and educational attainment.

## Discussion

Gender, when measured as a set of gender-distinct behaviours in adolescence, is associated with differential patterns of social mobility for adolescent males. In adolescence, more masculine-behaving male adolescents tend to have less advantaged backgrounds and do less well in school. These masculine-behaving male adolescents also tend to have lower levels of educational and occupational attainment 14 years later compared with their same-sex school peers. To test the implications of gendered context, we looked within schools at the quasi-random set of school-year peers an individual has. We found that male adolescents whose same-sex school-year peers are more masculine behaving than individuals in other years in their school are downwardly mobile, tending to stay in school for fewer years and having lower status jobs in early midlife. By contrast, female adolescents are insenitive to the gendered behaviour of their school-year peers.

We emphasise three important aspects of our findings. First, more masculine-behaving male adolescents are less likely than their schoolmates to end up in occupations with large proportions of female colleagues. This finding is important because, among the male respondents in Add Health, jobs that tend to have a relatively larger share of women tend to be of higher status. If men with masculine behaviour are less likely to join areas of the labour market where they are going to have many female coworkers, it might restrict their economic opportunities. Second, by early midlife, men with more masculine behaviour are downwardly mobile than their peers. They have less education, lower occupational status, and are more likely to have had contact with the criminal justice system. However, despite these empirical observations, they do not report feeling worse off when asked to place themselves on a respect ladder. This finding suggests that either the reduction in attainments are not salient (a possibility given that the observed magnitudes are noticeable at the population level but perhaps not salient at the individual level) or that they have chosen to trade economic returns for psychological returns (eg, they have chosen occupations that the public typically holds in high esteem, such as firefighting). Our findings regarding men align with those of others^29,30^ suggesting an urgent need for more research on this cohort. Third, attainments among female respondents were relatively insensitive to their gendered context. These results, along with those of others noting a pattern of female resilience,^31^ raise profound questions about our understanding of gender that merit follow-up work.

Our study has two important limitations. First, Add Health respondents were born in the late 1970s and had different environmental exposures than do contemporary adolescents, potentially restricting the generalisability of these results to adolescents nowadays. Second, our measure of gendered behaviour might not capture the relevant features of an individual’s environment with respect to gender for girls and women. For example, if US society during the adolescence of Add Health respondents had uniformly high levels of gender bias of a type that affected female adolescents, it might explain why we do not observe covariance of female attainment with our measure of gendered context.

Future work could use our framework to study gender norms in conjunction with the pursuit of gender equality emphasised in SDG 5^10^ and in the recent *Lancet* Series on gender equality, norms, and health.^32^ Given that our results suggest that gender norms are differentially relevant for men and women, several questions are raised. Research focusing on the relative resilience of women would be of great interest. Female attainments are relatively insensitive to their gendered context (as measured here)—is this true in general? Previous research on longevity has similarly suggested that women might be relatively resilient to the normative environment compared with men.^31^ Do women deploy coping mechanisms that could be adopted by men? By contrast, research into the mechanisms behind male sensitivity to gender norms would be informative. For example, what are the decisions that lead to reductions in educational and occupational attainment for men as a function of gendered context. If men in highly gendered contexts are largely choosing jobs that have reduced economic prestige but greater psychological value, it would perhaps suggest opportunities for messaging around occupational choices that could reduce gender segregation in some areas of the workforce. Answers to these questions could help improve our understanding of gender norms and their implications for men and women, but we would also suggest that measuring and monitoring gender norms and their associations with social mobility might be beneficial with respect to fostering progress on SDG 5. Gender norms provide information about what is expected from individuals with regards to gender performance, and monitoring their variation over time and place might offer important information about changes in the expectations and opportunities afforded to individuals.

### Contributors

BWD designed the study with input from BC, JMN, HBS, AMW, GLD, and KMH. BWD did the analysis and drafted the manuscript. All authors provided critical feedback on early versions of the manuscript. All authors read and approved the final version of the manuscript.

### Declaration of interests

We declare no competing interests.

## References

[1] Butler J. Performative acts and gender constitution: an essay in phenomenology and feminist theory. Theatre J 1988; 40: 519.

[2] Steinberg L. Cognitive and affective development in adolescence. Trends Cogn Sci 2005; 9: 69–74. 15668099 10.1016/j.tics.2004.12.005

[3] Chambers RA, Taylor JR, Potenza MN. Developmental neurocircuitry of motivation in adolescence: a critical period of addiction vulnerability. Am J Psychiatry 2003; 160: 1041–52. 12777258 10.1176/appi.ajp.160.6.1041 PMC2919168

[4] Resnick MD, Bearman PS, Blum RW, et al. Protecting adolescents from harm. Findings from the National Longitudinal Study on Adolescent Health. JAMA 1997; 278: 823–32. 9293990 10.1001/jama.278.10.823

[5] Shakya HB, Domingue B, Nagata JM, Cislaghi B, Weber A, Darmstadt GL. Adolescent gender norms and adult health outcomes in the USA: a prospective cohort study. Lancet Child Adolesc Health 2019; 3: 529–38. 31155319 10.1016/S2352-4642(19)30160-9 PMC6686658

[6] Viner RM, Ozer EM, Denny S, et al. Adolescence and the social determinants of health. Lancet 2012; 379: 1641–52. 22538179 10.1016/S0140-6736(12)60149-4

[7] Weber AM, Cislaghi B, Meausoone V, et al. Gender norms and health: insights from global survey data. Lancet 2019; 393: 2455–68. 31155273 10.1016/S0140-6736(19)30765-2

[8] Mollborn S, Domingue BW, Boardman JD. Understanding multiple levels of norms about teen pregnancy and their relationships to teens’ sexual behaviors. Adv Life Course Res 2014; 20: 1–15. 25104920 10.1016/j.alcr.2013.12.004 PMC4120999

[9] Mollborn S, Domingue BW, Boardman JD. Norms as group-level constructs: investigating school-level teen pregnancy norms and behaviors. Soc Forces 2014; 93: 241–67. 26074628 10.1093/sf/sou063 PMC4460822

[10] United Nations Transforming our world: the 2030 agenda for sustainable development. 2015 https://sustainabledevelopment.un.org/post2015/transformingourworld (accessed Oct 7, 2019).

[11] Fleming PJ, Harris KM, Halpern CT. Description and evaluation of a measurement technique for assessment of performing gender. Sex Roles 2017; 76: 731–46. 28630528 10.1007/s11199-016-0657-3 PMC5473164

[12] Nowotny KM, Peterson RL, Boardman JD. Gendered contexts: variation in suicidal ideation by female and male youth across U.S. states. J Health Soc Behav 2015; 56: 114–30. 25722128 10.1177/0022146514568350 PMC6097623

[13] Maselko J, Kubzansky LD. Gender differences in religious practices, spiritual experiences and health: results from the US General Social Survey. Soc Sci Med 2006; 62: 2848–60. 16359765 10.1016/j.socscimed.2005.11.008

[14] Vandello JA, Cohen D, Ransom S. US southern and northern differences in perceptions of norms about aggression: mechanisms for the perpetuation of a culture of honor. J Cross Cult Psychol 2008; 39: 162–77.

[15] Adler NE, Rehkopf DH. U.S. disparities in health: descriptions, causes, and mechanisms. Annu Rev Public Health 2008; 29: 235–52. 18031225 10.1146/annurev.publhealth.29.020907.090852

[16] Heise L, Greene ME, Opper N, et al. Gender inequality and restrictive gender norms: framing the challenges to health. Lancet 2019; 393: 2440–54. 31155275 10.1016/S0140-6736(19)30652-X

[17] Leaper C, Friedman CK. The socialization of gender In: Grusec JE, Hastings PD, eds. Handbook of socialization: theory and research. New York, NY: Guilford Press, 2007: 561–87.

[18] Kleinert S, Horton R. Adolescent health and wellbeing: a key to a sustainable future. Lancet 2016; 387: 2355–56. 27174303 10.1016/S0140-6736(16)30297-5

[19] Eccles JS, Roeser RW. Schools as developmental contexts during adolescence. J Res Adolesc 2011; 21: 225–41.

[20] Harris KM, Halpern CT, Whitsel EA, et al. Cohort profile: the National Longitudinal Study of Adolescent to Adult Health (Add Health). Int J Epidemiol 2019; published online June 29 DOI:10.1093/ije/dyz115. PMC6857761 31257425

[21] Dunn LM, Dunn L, Dunn D. Peabody picture vocabulary test. Circle Pines, MN: American Guidance Service, 1997.

[22] Riegle-Crumb C, Muller C, Frank K, Schiller K. Adolescent Health and Academic Achievement (AHAA). User’s guide and codebook, first release. Chapel Hill, NC: Carolina Population Center University of North Carolina at Chapel Hill, 2005.

[23] Belsky DW, Domingue BW, Wedow R, et al. Genetic analysis of social-class mobility in five longitudinal studies. Proc Natl Acad Sci USA 2018; 115: E7275–84. 29987013 10.1073/pnas.1801238115 PMC6077729

[24] Belsky DW, Caspi A, Arseneault L, et al. Genetics and the geography of health, behaviour and attainment. Nat Hum Behav 2019; 3: 576–86. 30962612 10.1038/s41562-019-0562-1 PMC6565482

[25] Peek CW, Lowe GD, Williams LS. Gender and God’s word: another look at religious fundamentalism and sexism. Soc Forces 1991; 69: 1205–21.

[26] Trejo S, Belsky DW, Boardman JD, et al. Schools as moderators of genetic associations with life course attainments: evidence from the WLS and Add Health. Sociol Sci 2018; 5: 513–40. 30613760 10.15195/v5.a22 PMC6314676

[27] Goldman-Mellor S, Caspi A, Arseneault L, et al. Committed to work but vulnerable: self-perceptions and mental health in NEET 18-year olds from a contemporary British cohort. J Child Psychol Psychiatry 2016; 57: 196–203. 26791344 10.1111/jcpp.12459 PMC4789764

[28] Chen P, Chantala K. Guidelines for analyzing Add Health data. Carolina Population Center University of North Carolina at Chapel Hill, 2014: 1–53.

[29] Coile CC, Duggan MG. When labor’s lost: health, family life, incarceration, and education in a time of declining economic opportunity for low-skilled men. J Econ Perspect 2019; 33: 191–210.

[30] Edin K, Nelson T, Cherlin A, Francis R. The tenuous attachments of working-class men. J Econ Perspect 2019; 33: 211–28.

[31] Cullen MR, Baiocchi M, Eggleston K, Loftus P, Fuchs V. The weaker sex? Vulnerable men and women’s resilience to socio-economic disadvantage. SSM Popul Health 2016; 2: 512–24. 10.1016/j.ssmph.2016.06.006 PMC5757782 29349167

[32] Gupta GR, Oomman N, Grown C, et al. Gender equality and gender norms: framing the opportunities for health. Lancet 2019; 393: 2550–62. 31155276 10.1016/S0140-6736(19)30651-8

